# Historical Sketch of Some of the London Hospitals.—III

**Published:** 1904-05-21

**Authors:** J. E. R. Stephens


### HISTORICAL SKETCH OF SOME OF THE LONDON HOSPITALS.—III.

By J. E. R. Stephens.

(*Continued from page* 379).

### Bethlehem Hospital.

Bethlehem Royal Hospital, vulgarly termed Bedlam, situated in the Lambeth Road, St. George's Fields, is a hospital for insane people, founded in Bishopsgate Without, and for a different purpose, in 1246, by Simon Fitz-Mary, one of the sheriffs of London. He founded it to be a priory of canons, with brethren and sisters, and to wear a star upon their copes and mantles, and to receive the Bishop of Bethlehem and the canons or messengers of the church of Bethlehem, whensoever they should have occasion to travel hither. The site of the original hospital was that known long after its removal as Old Bethlehem, subsequently as Liverpool Street. The greater part of it is now occupied by the stations of the North London and Great Eastern Railways. On the petition of Sir John Gresham (Lord Mayor), Henry VIII. gave the building of the dissolved priory, in 1547, to the City of London, in order that it might be converted into a hospital for lunatics. In 1557 the management was given to the governors of Bridewell Hospital.

This hospital stood in an obscure and close place, near unto many common sewers, and also was too small to receive and entertain the great number of distracted persons, both men and women. Therefore, upon a charitable consideration of the same, the Lord Mayor, Aldermen, and Common Council of the City of London, did grant unto the governors a sufficient piece of ground against London Wall on the south side of the lower quarters of Moorfields. And in pursuance thereof they proceeded to build a new hospital, containing in length 540 feet, and in breadth 40 feet. An inscription over the entrance stated that it was commenced in April, 1675, and finished in July, 1676—an instance of rapid building for those times. Robert Hooke was the architect, "the cost was nigh £17,000." In this as in other cases quick building did not imply sound building. When it was pulled down in 1814, it was discovered that the foundations were very bad, "it having been built on a part of the town ditch, and on a soil very unfit for the erection of so large a building." The inside consisted chiefly of two galleries one over the other, each 193 yards long, 13 feet high, and 16 feet broad, not including the cells for the patients, which were 12 feet deep. The said galleries were divided in the middle by two iron gates. So that all the men were placed in one end of the house, and all the women at the other, each having their proper conveniences, as likewise a store-room, where in the winter they had a fire to warm them.

By the beginning of the seventeenth century Bethlehem Hospital had become one of the London sights, and it so continued till the last quarter of the eighteenth century. At one time the hospital "derived a revenue of at least £400 a year from the indiscriminate admission of visitors." In 1770 it appeared at last to have dawned on the authorities that the practice "tended to disturb the tranquillity of the patients." The practice was continued for a few years longer, when it was put an end to, and no one was afterwards admitted without a particular introduction.

The first hospital could accommodate only 50 or 60 patients, and the second 150. By the end of the eighteenth century the hospital had become quite inadequate to the increased requirements. The City offered to grant a lease of some adjoining ground for its enlargement, but a committee appointed to consider what steps should be taken reported, April 1799, that the whole building was "dreary, low and melancholy," and the interior ill contrived, and further that it was in a very dilapidated condition. Eventually it was determined to remove the hospital to a more open situation, and the Bridge House Committee agreed to exchange a site of nearly 12 acres in St. George's Fields, Lambeth, for the two acres on which the hospital stood. An Act of Parliament was passed in 1810 sanctioning the exchange and removal. The Government granted a sum of £72,819 towards the necessary expenses, on condition that a wing of the new building should be appropriated to criminal lunatics; the City Corporation added £5,000; the City Companies and other public bodies contributed liberally; £5,700 was raised by public subscription. The first stone of the new building, designed by James Lewis, was laid April 18th, 1812, and it was completed in 1815, at a cost of £122,572. The new building provided accommodation for 198 patients, provision being made for future extension. Additions were made during the 25 years, 1843-1868, when Sidney Smirke was the architect.

The present object of this hospital is the reception of persons of unsound mind likely to recover within a year; also a certain number of incurables. All persons of unsound mind, presumed to be curable, eligible for admission, for maintenance and medical treatment, except those who have sufficient means for suitable maintenance in private asylums; those insane for more than twelve months, and considered by the resident physician to be incurable; those in a state of idiocy, or subject to epileptic fits, or whose condition threatens speedy death, or requires permanent and exclusive attendance of a nurse. Preference given to persons of the educated classes, to secure accommodation for whom no patient will be received who is a proper object for admission into a county lunatic asylum. A limited number of patients received on payment of £2 2s. per week, no social differences being made between patients on account of payment. The income in 1899 was £39,000, including £3,800 for incurables.

### London Hospital.

The London Hospital, Whitechapel Road, for the medical and surgical relief of the sick and injured poor, and especially workmen, seamen in the merchant service, and their wives and families, was instituted November 2nd, 1740, as the London Infirmary, in a large house in Prescot Street, Goodman's Fields (afterwards the Magdalen Hospital). But outgrowing the capacity of that building, a new site was purchased "in an airy situation, near the Mount in Whitechapel Road," and the first stone of a more capacious and more commodious building was laid on June 10, 1752, when the front portion was erected; it was added to from 1758 to 1760, when £18,000 was expended. Boulton Mainwaring was the surveyor, 1761–1771, when another £18,500 was expended. £23,000 was expended in 1781–83 under John Spillar. The West, or Alexandra Wing, was commenced on July 4th, 1864, the Prince of Wales laying the first stone; another wing, called the Grocers' Company's Wing—so named from the company having commenced the subscription for its erection with the handsome donation of £25,000 —was opened by the Queen on March 7th, 1876.

Accidents and urgent cases are admitted free at all times. No patient may remain longer than six weeks in ordinary, or two months in extraordinary cases unless by express permission. There is also a Hebrew ward for treatment of sick and injured persons of the Jewish faith. Attached as an appendage to the hospital is a most valuable institution called the Samaritan Society, which sends to a convalescent home, or provides with other suitable assistance, "domestic servants, mechanics, labourers, and others, who through sickness or accident have been obliged to quit their places to go into the hospital, and on being discharged have no home to go to or friends to receive them. It assists other discharged patients who have lost their furniture, or through sickness have been compelled to sell or pawn their clothes or tools; in cases of amputation supplies wooden legs, etc., and takes care that no patient who requires a truss shall leave the hospital without one. It also relieves families of patients during stay in hospital, and relieves poor married women with food, e[illegible] during confinement.

### Middlesex Hospital.

Middlesex Hospital, situated in Mortimer Street, [illegible] hospital for the reception and gratuitous treatment of si[illegible] lame, and cancer patients, originated in the year 1745, [illegible] the benevolent exertions of a few individuals. The hosp[illegible] consisted at first of a building in Windmill Stre[illegible] Tottenham Court Road, but soon a convenient site w[illegible] found in the Marylebone Fields, and a lease of 999 ye[illegible] was obtained from Mr. Charles Berners. The building, a[illegible] the design of J. Paine, architect, was commenced, the f[illegible] stone being laid, May 18, 1755. The building of the wi[illegible] was not completed until 1775. Enlargements were made [illegible] 1800, 1815, 1834, 1849, and 1859. The hospital was in[illegible] porated in 1836. Originally the funds could only supp[illegible] 18 beds, but means increasing in 1800, 70 beds were m[illegible] up; in 1815, 179; in 1824, 200; and in 1845, 250. Th[illegible] are three wards devoted to the treatment of females suffer[illegible] from cancer, named respectively "Whitbread," "Staffo[illegible] and "Laggan," after the benefactors, the first being endo[illegible] in 1792 by Samuel Whitbread. Large additions to [illegible] hospital have been made during the last few years wh[illegible] occupy a portion of the west side of Cleveland Str[illegible] The Medical College at the corner of Union Str[illegible] was opened in 1887 by Sir Reginald Hanson, Lord Ma[illegible] There is a convalescent home at Clacton-on-Sea for pati[illegible] from the hospital who require change of air before [illegible] course of treatment is concluded, or to recruit their stre[illegible] before resuming work. Provision is also made for nu[illegible] who from sickness or other causes require rest and cha[illegible] of air.

### St. George's Hospital.

St. George's Hospital, Hyde Park Corner, was founde[illegible] 1733 "for the relief of poor, sick, and disabled persons," [illegible] incorporated 1834. The hospital was designed 1828-2[illegible] William Wilkins, R.A., architect of the National Galler[illegible] the site of Lanesborough House, the London residen[illegible] Lord Lanesborough. Lanesborough House was conve[illegible] into a hospital for 60 patients in 1733, and on Janua[illegible] 1734, both in and out-patients were received. Large a[illegible] tions were made in 1859, under A. Mee, architect, at a [illegible] of £6,000; and again in 1868, on the south side, givi[illegible] lecture theatre for 200 students, and other necessary ac[illegible] modation. At Wimbledon is situated the Atkinson Mo[illegible] Convalescent Hospital for patients discharged from [illegible] George's Hospital. At St. George's Hospital, John Hu[illegible] the great surgeon, died (October 16th, 1793). He had [illegible] suffered from an affection of the heart, and in alterca[illegible] with one of his colleagues about a matter of right, w[illegible] had been by the Governors of the hospltal, as he tho[illegible] improperly refused him, he suddenly stopped, retired t[illegible] ante-room, and immediately expired.

